# Endometrial Cancer Immune Escape Mechanisms: Let Us Learn From the Fetal–Maternal Interface

**DOI:** 10.3389/fonc.2020.00156

**Published:** 2020-03-12

**Authors:** Valentina Bruno, Giacomo Corrado, Denisa Baci, Benito Chiofalo, Maria Antonia Carosi, Livia Ronchetti, Emilio Piccione, Adriana Albini, Douglas M. Noonan, Giulia Piaggio, Enrico Vizza

**Affiliations:** ^1^Gynecologic Oncology Unit, Department of Experimental Clinical Oncology, IRCCS—Regina Elena National Cancer Institute, Rome, Italy; ^2^Gynecologic Oncology Unit, Department of Women and Children Health, Fondazione Policlinico Universitario A. Gemelli, IRCCS—Università Cattolica del Sacro Cuore, Rome, Italy; ^3^Department of Biotechnology and Life Sciences, University of Insubria, Varese, Italy; ^4^Anatomy Pathology Unit, Department of Research, Diagnosis and Innovative Technologies, IRCCS—Regina Elena National Cancer Institute, Rome, Italy; ^5^Section of Gynecology, Academic Department of Surgical Sciences, Tor Vergata University Hospital, University of Rome “Tor Vergata”, Rome, Italy; ^6^School of Medicine and Surgery, University of Milano-Bicocca, Monza, Italy; ^7^Vascular Biology and Angiogenesis Laboratory, Science and Technology Pole (PST), IRCCS MultiMedica, Milan, Italy; ^8^Department of Research, Diagnosis and Innovative Technologies, UOSD SAFU, IRCCS—Regina Elena National Cancer Institute, Rome, Italy

**Keywords:** cancer immune escape, fetal–maternal immune tolerance, immunotherapy potential targets, immunological parallelism in cancer and pregnancy, personalized medicine

## Abstract

The immune escape mechanisms at the base of tumor progression in endometrial cancer mimic immune tolerance mechanisms occurring at the maternal–fetal interface. The biological and immunological processes behind the maternal–fetal interface are finely tuned in time and space during embryo implantation and subsequent pregnancy stages; conversely, those behind cancer progression are often aberrant. The environment composition at the maternal–fetal interface parallels the pro-tumor microenvironment identified in many cancers, pointing to the possibility for the use of the maternal–fetal interface as a model to depict immune therapeutic targets in cancer. The framework of cancer environment signatures involved in immune adaptations, precisely timed in cancer progression, could reveal a specific “immune clock” in endometrial cancer, which might guide clinicians in patient risk class assessment, diagnostic workup, management, surgical and therapeutic approach, and surveillance strategies. Here, we review studies approaching this hypothesis, focusing on what is known so far about oncofetal similarities in immunity with the idea to individualize personalized immunotherapy targets, through the downregulation of the immune escape stage or the reactivation of the pro-inflammatory processes suppressed by the tumor.

## Introduction

Innate and adaptive immune response affects development and progression of cancer through a process named immunoediting (1). Similar immune-mediated processes occur at the maternal–fetal interface (2–8). There is a parallelism between biological processes behind cancer progression and those behind the maternal–fetal interface such as proliferation, invasion, and angiogenesis (2). While these processes are finely tuned during embryo implantation stages, they are conversely often aberrant in carcinogenesis. Interestingly, a recent study based on single-cell analysis highlights that environment composition at the maternal–fetal interface parallels the pro-tumor microenvironment (TME) identified in many cancers (9), pointing to the possibility for the use of the maternal–fetal interface as a model to depict immune therapeutic targets in cancer.

**Figure 1:**
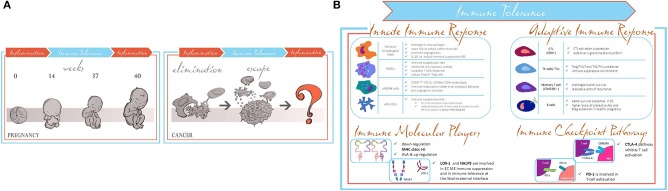
**(A)** Pregnancy and cancer parallelism in switching from activation to modulation of the immune responses. **(B)** Common shared immune tolerance patterns in pregnancy and EC.

Inflammation and immune tolerance are key mechanisms which ensure the proper establishment of pregnancy. The early stage of pregnancy is characterized by an inflammatory process responsible for proper implantation. This inflammatory stage should switch, in a second step, to a down-modulation of the immune response, ensuring the “non-rejection” of the semi-allogenic fetus. In pregnancy, the decidualized stromal cells, involved in the implantation process, are the gatekeepers of this key immune switching mechanism at the fetal–maternal interface, involving different immune cells, such as regulatory macrophages, natural killer (NK) cells, and T cells. In the last stage of pregnancy, and especially in activating labor, an inflammation process is required again; therefore, a new switching process is needed (10–18) (Figure 1A, left panel).

Several groups in the last years have shown that immunological properties acquired by both maternal–fetal interface and TME share the same molecular patterns related to the modulation of the inflammatory response involving innate and adaptive immune response (19). Tumor progression exploiting immune tuning mimics the immune maternal–fetal interface processes. In the early stage of carcinogenesis, the immune system recognizes cancer cells as non-self, inducing the proper pro-inflammatory environment to lead them to apoptosis. In a second step, the cancer cells are able to induce the switching of the immune system to an anti-inflammatory response, through different immune-editing mechanisms (20), thus leading to cancer immune escape. The fetal–maternal immune properties required to evade the immune system are limited in time and space; when labor starts, the immune system goes back to the initial steps. Conversely, the immune escape process in cancer goes on uncontrolled, and it does not revert to a pro-inflammatory feature (20) (Figure 1A, right panel). Therefore, the switching from immune suppression to immune activation occurring during pregnancy is lacking in carcinogenesis.

The existence of parallel situations between pregnancy and cancer gave rise to the term “oncofetal” and is common to many events. To take advantage of the knowledge of the similarities among immune regulation in pregnancy and tumor growth could lead to identification of new potential targets for cancer immunotherapy (2). Aberrations in placentation process, particularly in the modulation and tuning of the immune system, can lead to pregnancy complications; research has helped to develop the proper models to investigate immune tolerance in aberrant processes in pregnancy and to translate them to cancer investigations. Here, we summarize what is known so far about oncofetal similarities in immunity and which are the most recent and promising developments in this research area. A focus will be devoted to endometrial cancer (EC).

Therefore, this review is intended to focus on what is already known about the immune parallelisms between fetal–maternal interface immune tolerance and immune escape mechanisms during EC progression (Figure 1B).

## Focus on EC

EC is a relevant gynecological malignancy which occurs in fertile and postmenopausal women. The mean age of women affected by this tumor is decreasing; thus, the incidence in worldwide women under 40 years of age is on the rise (21). EC is linked to obesity (21), and the pandemic of obesity is a global threat. Patients with EC have a good prognosis at early-stage incidence, while the prognosis for recurrent or metastatic EC remains poor (22). It is therefore mandatory to understand the mechanisms fueling EC progression and ways to inhibiting them, to improve therapeutic chances (23). A deep knowledge of the interplay between positive and negative immunological molecular players and its timing in EC development and progression is still missing; several findings so far indicate that the immune escape mechanisms are at the base of EC progression and could be due to similar immune tolerance modulations occurring at the maternal–fetal interface (24). The EC is an ideal tumor model to study these mechanisms; EC tissue remains similar to the endometrial tissue of origin, and its related tumor progression develops in different steps, from endometrial hyperplasia to endometrioid carcinoma type 1 (grading: G1, G2, and G3), depending on its histological similarity to the physiological endometrial tissue (23). The immune escape pathways underlying the progression from physiological endometrium to carcinoma could represent new targets for personalized immunotherapy by the reactivation of the pro-inflammatory response processes suppressed by the tumor. A contribution to the immune escape in EC is also provided by the immunosuppressive interplay between regulatory T and regulatory B lymphocytes, regulatory NK, and tolerant dendritic cells, also traceable at the decidualized endometrial tissue in the fetal–maternal interface during implantation processes (24, 25).

The maternal–fetal interface and EC represent a “new” complex network where many immune cells of innate (dendritic cells, macrophages, and NK cells) and adaptive immune system cells (regulatory T and regulatory B lymphocytes) play a synergistic role in immune tolerance and immune escape mechanisms. The following part summarizes the similarities observed to date in the different cell lineages involved in the immunological processes underlying pregnancy and EC.

### EC Traditional Classification and Biomolecular Classification

EC was historically classified into two different clinicopathological and molecular types: type I is the much more common endometrioid adenocarcinoma (80–90%), and type II comprises non-endometrioid subtypes such as serous, clear-cell, and undifferentiated carcinomas, as well as carcinosarcoma/malignant-mixed Müllerian tumor (10–20%) (26). Molecular data are now considered as an important part of pathologic evaluation, since type I carcinomas are associated with PTEN, KRAS, CTNNB1, and PIK3CA genetic mutations and MLH1 promoter hypermethylation, whereas serous carcinomas show mostly TP53 mutations (27). Because of the limitations of this classification due to the wide molecular heterogeneity and, in turn, due to the discrepancy between the detected molecular pattern and tumor behavior, The Cancer Genome Atlas (TCGA) Research Network has gone further in the EC molecular landscape, providing more detailed molecular subclassifications, characterized, respectively by POLE mutation, mismatch repair deficiency, TP53 mutation, and a copy number low group without a specific driver mutation, each with a distinct prognosis (28): (i) POLE (ultra-mutated) tumors, (ii) microsatellite unstable (MSI) tumors, (iii) copy number high tumors with mostly TP53 mutations, and (iv) a remaining group without these alterations (29).

### Histopathologic Grades (G)

GX: Grade cannot be assessed.
G1: Well-differentiated.
G2: Moderately differentiated.
G3: Poorly or undifferentiated (28).

### FIGO Staging Classification

According to FIGO staging, EC is classified as below:

- Tumor confined to the corpus uteri
  IA No or less than half myometrial invasion
  IB Invasion equal to or more than half of the myometrium
- Tumor invades cervical stroma but does not extend beyond the uterus
- Local and/or regional spread of the tumor
  IIIA Tumor invades the serosa of the corpus uteri and/or adnexae
  IIIB Vaginal involvement and/or parametrial involvement
  IIIC Metastases to pelvic and/or para-aortic lymph nodes
    IIIC1 Positive pelvic nodes
    IIIC2 Positive para-aortic nodes with or without positive pelvic lymph nodes
- Tumor invades bladder and/or bowel mucosa and/or distant metastases
  IVA Tumor invasion of bladder and/or bowel mucosa
  IVB Distant metastasis, including intra-abdominal metastases and/or inguinal nodes (28).

### ESMO–ESGO–ESTRO Classification: Classes of Risk

The classification of risk groups defined in ESMO–ESGO–ESTRO consensus guidelines, comprehending a subdivision in low, intermediate, high-intermediate, and high risk, has been reached by a revision of the scientific literature within a consensus conference attended by a multidisciplinary panel of 40 experts. To sum up, these risk groups have been created by considering the clinicopathological prognostic factors which have an impact in identifying those patients who are at a higher risk of recurrence to properly address them to potential adjuvant therapies:

✓ Low-risk EC [stage I endometrioid, grades 1–2, <50% myometrial invasion, lymphovascular space involvement (LVSI) negative].
✓ Intermediate-risk EC (stage I endometrioid, grades 1–2, ≥50% myometrial invasion, LVSI negative).
✓ High-intermediate-risk EC (stage I endometrioid, grade 3, <50% myometrial invasion, regardless of LVSI status; or stage I endometrioid, grades 1–2, LVSI unequivocally positive, regardless of depth of invasion).
✓ High-risk EC (stage I endometrioid, grade 3, ≥50% myometrial invasion, regardless of LVSI status) (30).

## Molecular Players of the Immune Response

### Major Histocompatibility Complex: The Role of Human Leukocyte Antigens

Endometrial epithelial cells are potent antigen-presenting cells (APCs), while endometrial tumor cells show poor antigen-presenting capacity, leading to immune escape mechanisms. This poor antigen-presenting (AP) capacity is due to the downregulation of major histocompatibility complex (MHC) classes I and II in tumor cells and by the tolerogenic non-canonical MHC class Ib, human leukocyte antigen (HLA)-G upregulation (2, 24). Similarly, fetal trophoblast cells express tolerogenic rather than immunogenic MHC, the same HLA-G molecule, for its immune modulatory properties involved in the immune privilege condition in pregnancy. By a functional point of view, HLA-G expression on decidual stromal cells inhibits both innate and adaptive immune response. It mediates the inhibition of NK cell cytotoxic activity against trophoblast cells (31). Furthermore, it prevents cytotoxic T-cell capacities, enhances immune cell apoptosis processes, tunes cytokine secretion from blood mononuclear cells, and downregulates decidual stromal cell maturation and function (2). The expression of the same cell surface molecules could ensure immune privilege for both trophoblast at the maternal–fetal interface and cancer cells during tumor progression.

### Indoleamine-2,3-Dioxygenase

Tumor cells (including endometrial carcinoma cells) and tumor-infiltrating myeloid cells have a high amino acid consumption and therefore express high levels of indoleamine-2,3-dioxygenase (IDO) 1, an enzyme involved in tryptophan catabolism (2). Clinically, IDO is associated with myometrial invasion, lymph node metastases, lymphovascular space involvement, and poor survival. In cancer, APCs and IDO-expressing tumor cells seem to locally contribute to the immunosuppression state in tumor-draining lymph nodes (2, 24). Tryptophan breakdown by the enzyme IDO is one of the key mechanisms involved in fetal–maternal immune tolerance establishment. Furthermore, amino acid deprivation by IDO decreases the effector T cell/Treg ratio, leading to immunological tolerance. Thereby, IDO is involved in immune suppression processes which allow embryonic implantation or tumor growth. In particular, it is involved in tolerance promoting both the fetal–maternal interface and TME by counteracting lymphocyte proliferation (2).

### Galectins

Different studies on pregnancy pathologies show that galectins are expressed widely at the fetal–maternal interface (32). Galectins are a group of soluble β-galactoside binding proteins, consisting of 15 different types, that regulate crucial mechanisms of EC inflammation, tumorigenesis, and progression and are critical players of maternal immune tolerance (32). Notably, galectin-1 (33–35) has been proposed as a novel prognostic marker for EC. In addition, several immune cells such as decidual NK (dNK), macrophages, Tregs, and B cells, with essential roles in the establishment and maintenance of pregnancy, synthesize and respond to galectins (32). Galectins that have an important role in pregnancy are galectin-1 that regulates the expression HLA-G (36) and galectin-9 that interacts with T-cell immunoglobulin mucin domain-3 (Tim-3) receptor leading to Th1- and Th17-cell apoptosis and promoting immune tolerance. Tim-3–galectin-9 interaction facilitates the immunosuppressive activity of peripheral NK cells (37, 38) and promote decidual macrophage polarization to the M2 subtype (38).

### Lectin-Like Oxidized Low-Density Lipoprotein Receptor-1 (LOX-1)

Originally identified as an oxidized low-density lipoprotein (LDL) receptor, LOX-1 has gained attention for its role in the innate and adaptive immune response and has been correlated with immune suppression and tumor progression. LOX-1 has been indicated as a distinct surface marker for polymorphonuclear myeloid-derived suppressor cells (PMN-MDSC or granulocyte G-MDSC) (39, 40). LOX-1^+^ PMN-MDSCs inhibit T-cell function and proliferation, establishing immunosuppressive mechanisms in different cancers (41). In the same manner, LOX-1^+^ PMN-MDSCs may regulate immune tolerance at the maternal–fetal interface since reduced levels of PMN-MDSCs have been associated with miscarriage (42).

### NOD-Like Receptor Family, Pyrin Domain-Containing Protein 3 (NALP3)

NALP3 is an intracellular complex that regulates the innate immune activity through modulation of the production of pro-inflammatory cytokines. Dysregulation of NLRP3 inflammasome is implicated in tumor pathogenesis and cancer progression of EC and has been proposed as a new therapeutic target for EC (43, 44). NALP3 has also been involved both in the disruption of maternal–fetal immune-tolerance and in pregnancy complications (45–48). Thus, targeting the NLRP3 inflammasome can be essential to counter pregnancy dysfunctions and EC progression.

## Innate Immune Response Cell Infiltration

### Macrophages

Macrophages, phagocytic, and antigen-presenting innate immunity cells, are characterized by two polarization states, M1-like and M2-like. Tumor-associated macrophages (TAMs), which can be considered mostly as tolerogenic in tumor, are polarized into the two states depending on their location in the EC microenvironment (focal necrotic center of the tumor, which is hypoxic and triggers macrophage angiogenic function, or at the tumor margin), leading to anti-tumorigenic (M1) or pro-tumorigenic (M2) responses. The polarization and differentiation of macrophages into the cancer-inhibiting M1 and cancer-promoting M2 types represent a continuum of the two states of macrophages in the TME and influence disease progression and lymph node metastasis involvement. MHCII^high^ CD206^−^ TAMs are more M1 oriented and less able to penetrate hypoxic areas; conversely, MHCII^low^ CD206^+^ TAMs are more M2 oriented, able to invade hypoxic areas, promote angiogenesis, and even produce IL-10, a pro-tumorigenic immunosuppressive cytokine (24). IL-10-mediated immune tolerance is also crucial in pregnancy (49). An M2 polarization of decidual CD14^+^CD163^+^CD206^+^CD209^+^ macrophages is required for the maintenance of a physiological pregnancy, by exerting an immunosuppressive function (31, 49). Accordingly, M1/M2 unbalance is involved in pregnancy complications, such as preeclampsia and preterm delivery (31). In conclusion, a similar M1/M2 balance ensuring the proper immune privilege at the fetal–maternal interface is also used by the tumor, with a pathological purpose, to avoid the immune response.

### Myeloid-Derived Suppressor Cells

Two major MDSC subsets have been characterized based on their different phenotypes, the granulocyte G-MDSCs (CD14^−^CD15^+^CD66b^+^) and the monocyte M-MDSCs (CD14^+/−^CD15^low/−^). Most of the population identified in EC is the granulocytic type, which has a stronger immune suppression role (2, 24). Less is known about the role of the peripheral and decidual G-MDSCs and M-MDSCs in the successful pregnancy outcome (50). Peripheral MDSCs have an immunosuppressive role, by inhibiting NK-cell cytotoxic activity, T-cell proliferation, and Th2 polarization. By contrast, decidual MDSCs induce Foxp3^+^ Treg-cell proliferation and differentiation (50). Both mechanisms lead to the immunosuppressive state essential for the immune privilege acquired during pregnancy. It has been shown that in the first trimester, functionally suppressive peripheral and endometrial MDSCs are reduced in miscarriage patients, when compared with successful pregnancies (50). The lack of immunosuppression conferred by disrupted and MDSC function in pregnancy complications could represent a challenging mechanism to further investigate potential immunological targets for the EC cells to acquire a pro-inflammatory phenotype.

### Natural Killer Cells

The endometrial NK (eNK) cells in non-pregnant females appear to have a dedicated tissue-specific phenotype, different from NK cells in peripheral blood NK (pbNK) in the same donors (50). Uterine or decidual natural killer (dNK) cells in pregnant females have a CD56^superbright^ CD16^−^ CD49a^+^ CD9^+^ phenotype and represent an NK-cell subset that has been characterized within the developing decidua and constitutes ~50–70% of the lymphoid cells in the decidua in the first trimester (51). dNK cells are also involved in decidua and uterine spiral arteries and trophoblast invasion, by producing angiogenic cytokines and vascular endothelial growth factor (VEGF) and remodeling the maternal placental vascular bed, and are proangiogenic (51–54). dNKs have more immunomodulatory than cytotoxic behavior, by inducing the tolerogenic environment to host the fetus and permit the correct embryo implantation (6, 55) There are three types of subsets of dNK cells (dNK1, dNK2, and dNK3) in single-cell sequencing (9). The dNK1 cells express CD39 (an enzyme which is in the pathway to convert extracellular ATP into adenosine, which is an immunosuppressant), CYP26A1, B4GALNT1, HLA-G receptor, LILRB1, and higher expression levels of GZMA and GZMB mRNAs. The dNK2 cells are ANXA1 and ITGB2 positive, and dNK3 cells CD160, KLRB1, and CD103 express these antigens. dNK1 and dNK2 express activating NKG2C and NKG2E as well as inhibitory NKG2A, all of which are receptors for HLA-E molecules (9). This study finds no expression of VEGFA or IFNg by all three dNKs *in vivo*, in contrast with previous reports (52, 56, 57). However, they recruit extravillous trophoblast cells through CSF1, XCL1, and CCL5 (9). In repeated pregnancies, the dNK cells had more expression of NKG2C and LILRB1 compared to first pregnancies (57). Dysregulation in cytotoxic and regulatory NK-cell balance is involved in recurrent miscarriage and preeclampsia and pathogenesis mechanisms (51).

Peripheral blood and tumor-infiltrated NK cells in cancer patients express the CD56^bright^ CD16^−^ CD49a^+^ CD9^+^ phenotype (54, 58–61), and they have compromised cytotoxicity (54, 58, 59, 61–64). We have described a proangiogenic function in NK cells isolated from the peripheral blood and tumor-infiltrated cells of cancer patients (54, 58–60) and the expression of angiogenin, CXCR4/CXCL12, MMP2, MMP9, and tissue inhibitor for MMP (TIMP)-1 and TIMP-2 in NK cells from the peripheral blood and tumor-infiltrated cells of patients with colon cancer (59), which are molecules also secreted by dNK cells (55, 65–67). NK cells have been detected at low levels in the EC microenvironment (68); this study found significantly high levels of IL-1β and CXCL10 in the tumor, but low levels CXCL12, CCL27, and CCL21 as compared to the surrounding healthy tissue, and the NK cells in EC were compromised in cytotoxicity. The presence of NK cells predicts survival when HLA-E expression is upregulated; but it is associated with a worse prognosis when HLA-E expression is normal (69).

TGFβ is within the decidual microenvironment, and it has been shown to convert pbNK cells to a decidual-like phenotype (70–72), the treated cells express CD9 and CD49a on their surface, and they make VEGF (60, 72), and they also induce trophoblast invasion (72). TGFβ reduces the cytotoxicity of NK cells (73). Glycodelin-A is expressed in secretory endometrium, the decidua, and the amniotic fluid, which is involved in the maintenance of normal human reproductive activities. Glycodelin-A also converts CD56^bright^ CD16^−^ NK cells to dNK-like cells, which in turn regulate endothelial cell angiogenesis via VEGF and trophoblast invasion with insulin-like growth factor-binding protein 1 (74). The dNK cells also express glycodelin-A (75). TGFβ is a component of most TME (76–78), glycodelin is in various malignancies as in endometrial, ovarian, breast, lung and colon cancer (79).

### Dendritic Cells

In pregnancy, antigen-presenting CD83^+^ dendritic cells (DCs) are involved in Th-2 maintenance in decidual tissues through immunosuppressive cytokines secretion. It has been shown, in mice, that an impaired decidualization process, leading to embryo resorption, occurs in case of absence of uterine DCs. DC cells are also involved in angiogenesis impairment, through VEGFR1 and TGF-β1 pathways, which are key regulators of blood vessels maturation and endothelial cell survival (2). On the other side, although dendritic cells (HLA-DR^+^ DC), are increased in EC, these tumor infiltrating-DCs have lost their functions, with a reduced expression of costimulatory molecules, among which CD86, CD80, and CD40 (24). Their presence is adversely related to EC clinical features, such as clinical stage and lymph nodes metastasis (24). DCs play a key function in cancer not only for their immunoregulatory role but also since they produce angiogenic growth factors, thus leading to cancer progression (2). Therefore, DC impairment is involved in immune tolerance processes both in pregnancy and in EC: pregnancy complications, in which a disruption in the immune modulation is lacking, offering a model to further investigate future immunological perspectives in EC immunotherapy.

### Neutrophils

During immune responses, neutrophils are among the first cells to reach the site of inflammation. The increased number of tumor associated neutrophils is linked to poor outcomes in different type of cancers, and many patients with advanced EC show high levels of blood neutrophils (80–82). Several evidence suggest that cancer cells may manipulate neutrophils, sometimes early in their differentiation process, to create diverse phenotypic and functional polarization states able to alter tumor behavior. A polarization similar to that of macrophages has been suggested for neutrophils with a division into antitumorigenic neutrophils (N1) and protumorigenic neutrophils (N2) (83–86). It has been shown that various tumors are capable to predispose circulating neutrophils to produce neutrophil extracellular traps (NETs) causing systemic thrombosis which is often associated with human cancers (4, 87–89). Formation of NETs results in extrusion of nuclear and mitochondrial DNA mixed with granular and some cytoplasmic constituents, as neutrophil elastase (NE), myeloperoxidase (MPO), and the citrullination of histone H3 (CitH3), in response to infection or cancer burden (90). A switch in neutrophil phenotype toward a low-density neutrophil type (LDN) with more immature appearance and less lobulated nuclei has been found during tumor progression (91–94). An important feature of LDN is the capacity to form NETs. Furthermore, NETs have been reported to be composed by neutrophil derived matrix metalloproteinases (MMPs) and facilitate tumor invasion (95). Several studies have focused on the role of NETs in tumor development including tumor growth, metastasis, and angiogenesis (91, 95–99). NET structures can often be detected in proximity to human tumors and a large number of LDN have been found into the blood and in postoperative lavages in advanced cancers (92, 98). Taken these facts into consideration, NETs are supposed to facilitate tumor invasion providing a favorable microenvironment for the survival of trapped tumor cells. The mechanism of the release of the chromatin by stimulated neutrophils, called NETosis, may unveil unexpected functions of neutrophils in cancer development and can provide another explanation for the elevated circulating cell-free DNA (cfDNA) release in blood stream in pathologic conditions. We have recently demonstrated the potential of cfDNA as a simple and inexpensive tool to better adapt surgical staging and help EC stratification, and furthermore that the evaluation of cfDNA content maybe correlated with NETosis activation in EC (80, 100).

As previously described, neutrophils play a role in tissue remodeling and adaptation, according to the influence of sex hormones, in post mating inflammatory response (101), as well as in NETs-induced sperm trapping and clearance (102, 103).

In physiological pregnancy neutrophils cells switch to a mainly pro-inflammatory phenotype (104). It has been assessed *in vitro* that placental derived micro-debris could activate neutrophils and induce the NETs processes, as well as IL-8, a pro-inflammatory placentally derived cytokine (105).

These findings have been also confirmed in an *in vivo* setting: neutrophil NETs have been also described in physiological pregnancy at the placental intervillous space. The placenta physiologically release micro-debris, which could activate neutrophils and, in turn, the NETosis process. These findings are in line with the pro-inflammatory environment required in normal pregnancy. NET appears to be enhanced in preeclamptic placentae, up to involve the entire intervillous space. Furthermore, in preeclamptic rather than in normal pregnancies, the neutrophils which cross the utero-placental circulation show a more highly activated phenotype compared to the peripheral counterpart (106). Some further data suggest that NETs could be involved also in other pregnancy complications, such as intra-uterine growth restriction, preterm labor (107) and recurrent pregnancy loss, in which the auto-antibodies activation of neutrophils (108) might play a role in NET processes.

In conclusion, neutrophils through the recently investigated NET/NETosis processes, have a key role in both physiological adaptation of the endometrium ranging from fertility and pregnancy modifications and pathologic conditions such as EC, revealing a potential field of further investigation to target specific immune changes occurring in EC etiopathogenesis.

## Adaptive Immune Response Cell Infiltration

### T Cells

T cells-mediated adaptive immunity could have a positive role in terms of anti-tumorigenic effects. The key players in this response are APCs, among which macrophages, DC and B cells, which are able to present tumor antigen to CD4^+^ helper T cells. They in turn are polarized to Th1 response having a role in CD8^+^ T cells and B cell induction and in NK and macrophage activation. These pathways eventually activate tumor cell elimination by a CD8^+^ cytotoxic T cells (CTL)-dependent apoptosis (20). However, T-cell mediated response could also play a negative role in cancer progression: tumor cells and their released soluble factors are able to inhibit DC function, leading to a tolerogenic APC phenotype and to a suppression of CTL activation. Furthermore, the Treg/Th1 unbalanced ratio lead to an immune suppressive environment and to tumor escape. These steps make the possible pirating of immune checkpoint molecule pathways by cancer cells (20). CD8^+^ tumor infiltrating lymphocytes (TIL) in EC show defective granzyme B and perforin expression, leading to a lack of tumor-induced suppression activity. Clinically, increased CD8^+^ TIL at the tumor-invasive margin improved prognosis and their intra-tumor counterpart, associated with an improved disease-free survival in type I and II EC, have been found more frequently in low-grade than in high grade tumors. The presence of CD45RO^+^ memory T cells associates with overall survival (OS) and reduced events of recurrence. An elevated number of tumor Treg correlates with increased vascularity, tumor grade and stage, extent of lymph node metastases and myometrial invasion, worse disease-free survival. The resulting high Treg/CD8 and Treg/CD4 ratios it has been increased also in distal tumor-draining lymph nodes (TDLN). Conversely, proximal TDLN show increased CD4/CD8 ratio (24). In general, Treg cells in cancer are involved in blocking antitumor immunity, by suppressing effector T lymphocyte proliferation and enhancing tumor blood vessel density (2). Furthermore, the presence of the infiltrate consisting of lymphocytes and plasma cell as potentially tertiary lymphoid structures, is related to low-grade endometrial tumors, associated with favorable prognosis (24).

Concerning T cell function at the maternal–fetal interface, Th1/Th2 and Th17/Treg ratios are slightly tuned thorough all pregnancy. In the early stage a pro-inflammatory Th1 microenvironment is required to allow a successful implantation process. This response is then followed by a shift to Th2 immune phenotype (31). Placental tissue induces CD25^high^CD127^low^Foxp3^+^ Tregs expansion, which expressed suppressive markers, such as CTLA-4 and minimizes excessive Th cell activation, by decreasing Th1-, Th2-, and Th17-associated cytokines (109). CTLA-4 expression on Tregs enhances production of IDO in decidual DCs and monocytes. IDO, which is involved in fetal-maternal interface immune tolerance, limits T cells tryptophan availability in uterine microenvironment, thus modulating maternal T-cell activations (31).

An unbalanced Th1/Th2 and/or Th17/Treg ratios have been associated to several pregnancy complications, including preeclampsia and recurrent miscarriage (2).

In conclusion, Th1/Th2 and Th17/Treg ratios could be recognize as parallel features in immune-mediated processes, shared by both fetal-maternal immune tolerance establishment and cancer progression. Interestingly, Th17 and Treg cells show a wide extent of plasticity in different “inflammatory frameworks,” so that they can switch one another (110) thus opening the possibility for these ratios to be therapeutically actionable targets.

### B Cells

B cells behavior in EC environment has not been fully elucidated yet: a few information is available regarding their role in EC progression. However, it has been shown that co-presence of T and B cells in TME correlates with better survival outcomes (24). Higher production of protective antibodies occurs in healthy pregnancy. Beyond this well-known paradigm, there is an enhanced CD19^+^CD24^hi^CD27^+^ Breg expansion, which have regulatory function on immune responses. Briefly, B10, a subtype of Breg, cell subtypes are potent producers of IL-10 whose pathway is a key modulator in autoimmunity responses. IL-10 plays a key role in maintaining DCs in an immature state during pregnancy, inhibiting T cells activation and Th1 differentiation pattern. Lower CD19^+^CD24^hi^CD27^+^ Breg percentage in the first pregnancy trimester has been detected in women affected by miscarriages (49). This immune tolerance disruption could decipher molecular pathways deserving to be investigating in immune escape in EC.

## Immune Checkpoint Molecule Pathways

Immune checkpoint molecules are cell surface receptors involved in the negative regulation of immune response during infections, autoimmunity response, transplantation, tumor immune escape as well as at the maternal–fetal interface. CTLA-4 (cytotoxic T-lymphocyte-associated protein), TIM 3 and PD-1 are the most studied members. The physiological role of these molecules is to prevent immune attack against self-antigens during an immune response by inducing T cell exhaustion (111, 112).

As already describe above, pregnancy is a model of immune tolerance establishment, thus it represents a great immune challenge to further investigate immune checkpoint molecule roles in similar immune avoiding-based pathologies progression, such as cancer.

### CTLA-4

CTLA-4 is involved in EC immune escape processes, since its expression in Treg cells has a negative immune modulator role. By a mechanistic point of view, it binds the B7.1 and B7.2 ligands expressed on the APC surface. This binding inhibits, in a competitive way, the binding to CD28, thus preventing the activation of cytotoxic T lymphocytes (113).

During pregnancy, a high level of CTLA-4 expressing-Treg, DCs and monocytes expressing CTLA-4 B7.1 and B7.2 ligands are also present in the decidua. These data suggest that the same competitive bindings observed in EC are in place also during pregnancy. The increased presence of CTLA4 and its ligand at the decidua is positively correlated with Th2 cytokines secretion and negatively with Th1 cytokines production (114), pointing out the CTLA4 remarkable role on local immune-suppression effect. CTLA-4 is also involved in pregnancy complications, such as recurrent pregnancy loss and preeclampsia (115, 116), highlighting the hypothesis to use them as immune disruption challenging models, to be translated in EC cancer.

### PD-1

PD-1/PDL-1 pathway is involved in EC progression, EC cancer cells have on their cell surface the immune checkpoint molecules, therefore they can exert an antitumor immunosuppressive function. PD-L1 is expressed on cancer cells and on immune APCs: by binding PD-1, which is upregulated on the T cell surface, it can reduce proliferation and function of T cells, and induce apoptosis through Bcl-2. Among B7-H family, PDL-1 (B7-H1) is expressed in tumor endometrial cells at the same levels compared to normal endometrial cells; however, in PD-L1^+^ tumors, a trend in decreased survival has been shown. B7-H3 and B7-H4 expression pattern negatively correlates with TIL number (both whole T cell population and CTL one). For instance, B7-H4 has been shown to be upregulated in EC (2, 24).

On the maternal side, PD-1 is highly expressed by decidual T lymphocytes (its expression is induced by decidual CD8^+^, CD4^+^, and Treg cells), decidual stromal cells (its expression is enhanced by Th1 cytokines) and decidual macrophages. Conversely, PD-L1 and PD-L2 are expressed by trophoblast cells first, and then by placental tissues. PD-1 / PD-L1 interaction downregulates CD4^+^ T cells Th1 cytokine secretion, by contributing in the maintenance toward a Th2 shift immune balance at the maternal–fetal interface. PD-1/PD-L1 pathway dysregulation is involved in preeclampsia (increase PD-L1 expression in Th17 cells and higher PD-1 expression in Treg cells) and recurrent miscarriage (decreased decidual PD-L1 expression) (114).

### TIM-3

In EC, NK cells have a great variability in the expressed co-inhibitory molecule TIM-3, suggesting a correlation between its expression and the stage of the disease. It has demonstrated that TIM-3 expressing- NK cells are present at higher levels in patients with lymph node (LN) invasion, compared to patients with no LN involvement (68). Thus, an increasingly important immunosuppressive phenotype in NK cells is related to EC stage, involving in particular a correlation with LN metastases detection (68).

The decidual NK cells (dNK) 60–90% express TIM-3. TIM-3^+^ dNK cells show a phenotype switching to a Th2 cytokine profile, which is also expressed by decidual stromal cells (DSCs): higher levels of Th2 cytokines are produced by DSCs expressing TIM-3. Furthermore, TIM-3 activation seems to exert an anti-apoptotic function on DSCs under TLR stress signals (117). According to these findings, TIM-3 seems to regulate an exaggerated aberrant immune inflammatory response at the maternal–fetal interface, ensuring tolerance establishment (118). Decreased percentage of TIM-3 expressing- dNK cells and DSCs have been shown in recurrent pregnancy loss patients (38). Conversely an upregulation of TIM-3 has been identified in decidual tissues of preeclamptic women (119). These two pathological pregnancy immune conditions, suggest a disrupted Th1 immune response regulation, due to TIM-3 pathway.

## Endocrine-Immunological Fluctuation in Pregnancy and EC

The endometrial composition of the immune cells varies according to the hormonal changes within the menstrual cycle. Ranging from the follicular to the secretory phase hormonal changes, a proliferation in NK cell and macrophage populations occurs, with an increasing size in lymphoid aggregates made of a B cell core, surrounded by T cells (mostly memory T effector CD8^+^CD45RO^+^ cell) and margin consisting of macrophages. These aggregates, as well as the dendritic cells, are recruited to the endometrium. This hormone-immune fluctuation is bridging to address their function in the breakdown of the endometrium. Furthermore, antigen presentation processes are enhanced through CD40, CD1d, MHC I/II higher expression. During the secretory phase, an increase in Treg sub-population and in IDO expression occurs to allow a potential embryo implantation process, by leading to an immune tolerance microenvironment at the fetal-maternal interface (24). The T cell cytotoxic capacity is hormonal related, varying according to the different phases of the menstrual cycle, to ensure a correct balance between immune -protection and -tolerance (120). Neutrophils play mainly a key role in endometrial tissue remodeling both during the follicular phase, when the endometrial tissue became receptive to embryo implantation under the influence of the estrogen stimulation (121, 122) and during the secretory phase when neutrophils are located in tissue degradation areas, just before the menstruation occurs (123).

These endocrine-immunological fluctuations reveal important consequences also in the oncological condition: for instance, NK levels, which normally increase during menstrual cycle, are presents in low levels in the EC with a decreasing activity in advanced stages. Conversely, their levels increase upon progestin treatments in the conservative management of uterine carcinoma, leading to an oncological outcome improvement (124). These findings are in line with the higher NK levels during the secretory phase in the physiological condition, when progesterone levels are increased. Taken together, these findings support the idea that the physiological endocrine-immunological capacities of specific cell types which contribute to the fetal maternal tolerance environment, play also a key role in the tumor immune escape processes.

## Pregnancy and EC Shared Interplay Between the Immune and Angiogenesis Stimuli

Angiogenesis is the formation of new blood vessels from existing ones and occurs in most of the body's physiological processes, including fetal development, menstruation cycle, and wound repair (125). Proper placental angiogenesis starting at day 21 after conception and continuing throughout human gestation is of central importance to ensuring a healthy pregnancy (126, 127).

Within the decidual environment, the immune cells establish immune tolerance and a state of mild systemic inflammation important for the activation of vascular endothelium and angiogenesis. The rich immune infiltrate is also a major source of angiogenic factors that induce vascular growth (128, 129). Angiogenic factors such as VEGF (130), placental growth factor (PlGF) (126), fibroblast growth factor (FGF2) (131), MMPs, and TIMPs (132) are specifically and highly expressed in endothelium and placenta during pregnancy and play a synergic role to ensure the formation of the vascular network.

Angiogenesis is also recognized as one of the hallmarks of cancer and plays important roles in the growth and metastasis of EC (133, 134). Highly secreted proangiogenic factors in the fetal and maternal compartments such as VEGF, PlGF, and bFGF have been also associated with EC progression and metastasis (135–137). In particular, overexpression of VEGF has been correlated with advanced stage, high tumor grade, deep myometrial invasion, lymphovascular infiltration, lymph node metastases, and poor clinical outcome in patients with EC (138).

Pregnancy and cancer represent two dynamic conditions unique for the complex and finely tuned interplay between inflammation, tolerance, and angiogenesis. As previously discussed, the immune cell infiltration within the maternal–fetal interface or TME that would inherently be conceived as “foes” can transform into “friends” providing immune tolerance and supporting angiogenesis through the release of cytokines, growth factors, and chemokines necessary for placentation or tumor growth. For instance, M2 decidual polarization is associated with secretion of proangiogenic cytokines, such as VEGF, CXCL8, and MMPs (139). A positive correlation between the concentration of M2-oriented TAMs, angiogenesis, and advancement of EC has also been reported (140). Similarly, neutrophils maintain tolerance, protect against pregnancy complications (141, 142), and exert angiogenic features through a direct secretion of cytokines like VEGF and CXCL8 (143). In EC, a dualistic angiogenic and mitogenic role of TAN cytokines has been observed (144). It has been suggested that failure in antiangiogenic treatment based on anti-VEGF therapy can be correlated with TAN activities (145).

Another excellent example within the uterine environment is NK cells “tuning” into dNK, which represent a major source of angiogenic factors supporting placentation (52). On the other hand, EC TME reshapes NK-cell phenotype and function to promote tumor progression (68) and acquire a decidual-like and proangiogenic phenotype/function (53, 59, 146) in patients with various cancers.

Within the decidual environment, the dual role of the immune cells ensures immune tolerance and angiogenesis, highlighting the importance of the fetal–maternal immunologic dialogue in a normal pregnancy. Similarly, cytokines, growth factors, and chemokines released by immune cells can contribute to an immunosuppressive TME and stimulate both angiogenesis and tumor progression.

Several antiangiogenic drugs, used either alone or in combination with chemotherapy, have presented mixed results in treating EC patients. Failure in the treatment of vascularized gynecological cancers, including EC, which are mainly focused on targeting cell cycle and angiogenesis, could be better managed and improve the antitumor activity by combining immune checkpoint blockade and antiangiogenic therapies (147, 148). Antiangiogenic therapy could block VEGF–VEGFR2 axes and downstream signaling pathways, resulting in normalization of tumor blood vessels and consequently enhancing T-cell recruitment and increasing T-cell infiltration into the TME. Combined therapy of lenvatinib (multikinase inhibitor of VEGFR1, VEGFR2, and VEGFR3 and other receptor tyrosine kinases) plus pembrolizumab (an antibody targeting PD-1) showed antitumor activity in patients with advanced recurrent EC (149).

Understanding the immunoregulatory network during pregnancy will help identify new targets of immunotherapy and provide new avenues for immunologic reconstitution and angiogenic inhibition in patients with EC. Lymphangiogenesis, expansion of new lymphatic vessels from pre-existing ones, has also been observed at the maternal-fetal interface (150). However, there is a paucity of data and conflicting reports both on the lymphatic profile during pregnancy (151) and lymph vessel space involvement in different subsets of EC (152, 153).

## From Immune Suppression to Immune Activation in Labor: a Promise in Cancer?

Labor is characterized by a backtrack from the pregnancy-induced fetal–maternal immune tolerance framework toward a most likely inflammatory process. Both innate and adaptive immune systems are involved in labor onset (154). The innate immune response, through a switching in the polarization of monocyte and neutrophil cell phenotypes, could ensure proper tissue remodeling processes after birth. Both adaptive and innate immune systems are therefore involved in labor onset (154). The adaptive immune response is characterized by an increase in naive Treg phenotypes associated with a decreased function of Treg suppression of cell-mediated immune responses toward maternal non-self-fetal antigens (155). These changes are supported by the enhanced immune responsiveness both in the peripheral blood and in the local environment where an increase in monocyte subset proportions as well as a decline in Treg repression and a higher CD4 T-cell expression of MHC class II molecules occur, respectively. In summary, in labor onset, the peripheral naive Tregs increase, linked to the Treg function decrease (155, 156), which leads to a shift toward the Th1-like and Th17 responses, normally suppressed during pregnancy. Therefore, in this last stage of pregnancy, a Th2 repression occurs. In conclusion, maternal–fetal immune tolerance is lost in labor both peripherally and locally, in favor of a more pro-inflammatory framework (157, 158) supported by enhanced pro-inflammatory cytokine secretions such as IFN-γ, TNF-α, IL-6, and granulocyte–macrophage colony-stimulating factor (GM-CSF) (154).

The switching from immune suppression to immune activation occurring during labor is lacking in carcinogenesis. However, lessons from labor would help us identify possible targets to avoid the immune escape of cancer cells or to switch from immune tolerance to inflammation environment that is missing in cancer which remains in the immunological tolerogenic state.

## Clinical Implications of Different Immunogenicities According to EC Biomolecular Classification, FIGO Stage, and ESMO–ESGO–ESTRO Classification, Including Risk Factors

To summarize, from a clinical point of view, high levels in CD8^+^ T cells have been shown to be independent positive prognostic factors in EC-related OS, especially in type I ECs, since a high CD8^+^ T cell/Treg cell ratio has been associated with low histological grading, early stages, superficial myometrial invasion, absence of lymphovascular space invasion (LVSI), lack of distant metastases, and lymph node involvement. Conversely, a higher Treg cell/CD8^+^ T cell ratio is associated with EC advanced stages, poorly differentiated grading, and LVSI. Therefore, disease-free survival (DFS) in these patients is significantly lower. Furthermore, CD3^+^ and CD8^+^ TIL cell counts are much higher in POLE ultra-mutated and MSI hyper-mutated tumors, with a higher tumor antigen load and an upregulation in PD-1 and PD-L1 expressions: a related strengthened immune activation could be responsible for their better prognosis compared to microsatellite stable EC (159, 160). TAM presence is mostly associated with LVSI, myometrial invasion, and lymph node metastases, showing a proangiogenic phenotype. Furthermore, a higher density in tumor immune infiltrate has been correlated with advanced FIGO stage and higher tumor grade, with a trend in lower recurrence-free survival (RFS) outcome (160).

PD-1 overexpression in both tumor-infiltrating and peritumoral lymphocytes of POLE-ultra-mutated and hyper-mutated/microsatellite unstable EC tumors suggests a potential application of a targeted immunotherapy, acting on the PD-1/PD-L1 pathway (161, 162).

Pembrolizumab, which is a humanized IgG4 monoclonal antibody against PD-1 (163), obtained FDA approval and, in patients affected by EC, showed progression after the recommended conventional therapy without any other available treatment approaches (164). The highest efficacy level of this kind of immunotherapy has been shown in MSI-high patients, in which specific microsatellite sequences are more subjected to copying errors in case of a compromised mismatch repair (MMR) mechanism. Also, in POLE ultra-mutated tumors associated with a high mutational burden, clinical beneficial effects have been shown, after pembrolizumab administration (165). To sum up, immunotherapy could open new personalized therapeutic approach strategies, once biomolecular predictive markers will be clearly identified in those EC patients who are at higher risk of recurrence or metastatic disease.

## Future Perspectives

To use the knowledge in fetal–maternal immune tolerance mechanisms and translate it into cancer immune-skipping processes could be a strength and a potential cornerstone to read the same problem from a different perspective, leading to promising and unexpected results. To study what in nature has been already built with a precise limitation in time and space, such as in the fetal–maternal immune tolerance, could lead to the recognition of these pathways also in processes not finely tuned, such as cancer immune escape. If we read research as re-search, this meaning could be to search again what already exists.

To know the exact composition of the intra-tumor immune infiltrate might influence treatment choice and application, as well as its outcome. Beyond the already well-known potential EC immune target therapy (with related potential application of IDO inhibitors, immune checkpoint inhibitors B7-H4 monoclonal antibodies, MDSC and all transretinoic acid, and NK-cell therapy), this parallel study setting could offer further future applications. A multi-omics approach (genomics, transcriptomics, proteomics, and immunomics) coupled with imaging approaches (digital pathology), customized on maternal–fetal immune tolerance signature and to be analyzed in the different tumor progression stages, could lead to the building of a network in which immunological changes could be identified at each time point.

As mentioned before, the immune adaptations through all pregnancy have been shown to be precisely timed also at a systemic level, outlining an immune clock of pregnancy. To arrange the immunological events into a chronological framework of pregnancy may unravel the immunological aberrations involved in pregnancy complications.

This concept implies that the immune adaptation signature to tumor progression could involve also systemic modifications, and it becomes crucial to study them in the peripheral environment. By using mass cytometry and Luminex, the percentage and functions of immune cell subsets, together with cytokine and chemokine cancer environment signatures, respectively, will be detected in peripheral blood samples, collected for each step of EC progression. The framework of cancer environment signatures involved in immune adaptations, precisely timed in cancer progression, could reveal a specific “immune clock” in tumor, and specifically in EC, which might be identified through a liquid biopsy.

## Conclusions

Based on the above considerations, the immune escape mechanisms at the base of tumor progression could be due to similar immune tolerance mechanisms occurring at the maternal–fetal interface: studies approaching this hypothesis could allow us to individualize personalized immunotherapy targets, through the downregulation of the immune escape stage or the reactivation of the pro-inflammatory processes suppressed by the tumor.

To unravel the precise timing of immunological events occurring during EC progression could have some clinical implications. Their clinical relevance resides on the possibility to stratify patients before and during treatments according to the immunological patterns observed on patient endometrial biopsies, leading to a personalized approach. It can be speculated that if we are able to modulate the immune system at the beginning of the immune-skipping process, this will be more effective than to try to modulate only specific single targets, when the immune process has already started.

To go further, using the immune tolerance system operating during pregnancy progression as a model system could be advantageous to identify molecular mechanisms responsible for the cancer immune escape. The final goal could be, as mentioned before, to orchestrate them in order to downregulate the cancer immune escape process and/or re-activate the last step of the pro-inflammatory response.

## Author Contributions

VB, LR, and GC conceived and designed the review. VB, DB, DN, and GP wrote the review. BC, MC, LR, and EP contributed in discussing the EC clinical aspects. DB, AA, and DN were responsible for NK and angiogenesis sections and revised the entire review. GP and EV coordinated all review realization steps, revised the review draft, and contributed to critical discussion.

### Conflict of Interest

The authors declare that the research was conducted in the absence of any commercial or financial relationships that could be construed as a potential conflict of interest.
